# Diagnostic challenge in a series of eleven patients with hyper IgE syndromes

**DOI:** 10.3389/fimmu.2022.1057679

**Published:** 2023-01-10

**Authors:** Roukaya Yaakoubi, Najla Mekki, Imen Ben-Mustapha, Leila Ben-Khemis, Asma Bouaziz, Ilhem Ben Fraj, Jamel Ammar, Agnès Hamzaoui, Hamida Turki, Lobna Boussofara, Mohamed Denguezli, Samir Haddad, Monia Ouederni, Mohamed Bejaoui, Koon Wing Chan, Yu Lung Lau, Fethi Mellouli, Mohamed-Ridha Barbouche, Meriem Ben-Ali

**Affiliations:** ^1^ Laboratory of Transmission, Control and Immunobiology of Infections, Institut Pasteur de Tunis, University Tunis El-Manar, Tunis, Tunisia; ^2^ Faculty of Medicine, Tunis El Manar University, Tunis, Tunisia; ^3^ Department of Pediatrics, Ben Arous Hospital of Tunis, Tunis, Tunisia; ^4^ Department of Pediatrics, National Bone Marrow Transplantation Center, Tunis, Tunisia; ^5^ Pulmonology B Department, AbderrahmenMami Hospital, Ariana, Tunisia; ^6^ Department of Dermatology, HédiChaker Hospital of SFAX, Sfax, Tunisia; ^7^ Department of Dermatology, Farhat Hached Hospital, Sousse, Tunisia; ^8^ Department of Pediatrics, Children Hospital of Tunis, Tunis, Tunisia; ^9^ Department of Pediatrics and Adolescent Medicine, Li KaShing Faculty of Medicine, The University of Hong Kong, Hong Kong, Hong Kong SAR, China; ^10^ Department of Microbiology, Immunology and Infectious Diseases, College of Medicine and Medical Sciences, Arabian Gulf University, Manama, Bahrain

**Keywords:** STAT3 deficiency, DOCK8 deficiency, differential diagnosis, candidate gene strategy, WES

## Abstract

Hyper IgE syndromes (HIES) is a heterogeneous group of Inborn Errors of Immunity characterized by eczema, recurrent skin and lung infections associated with eosinophilia and elevated IgE levels. Autosomal dominant HIES caused by loss of function mutations in Signal transducer and activator of transcription 3 (*STAT3*) gene is the prototype of these disorders. Over the past two decades, advent in genetic testing allowed the identification of ten other etiologies of HIES. Although Dedicator of Cytokinesis 8 (DOCK8) deficiency is no more classified among HIES etiologies but as a combined immunodeficiency, this disease, characterized by severe viral infections, food allergies, autoimmunity, and increased risk of malignancies, shares some clinical features with STAT3 deficiency. The present study highlights the diagnostic challenge in eleven patients with the clinical phenotype of HIES in a resource-limited region. Candidate gene strategy supported by clinical features, laboratory findings and functional investigations allowed the identification of two heterozygous *STAT3* mutations in five patients, and a bi-allelic *DOCK8* mutation in one patient. Whole Exome Sequencing allowed to unmask atypical presentations of DOCK8 deficiency in two patients presenting with clinical features reminiscent of STAT3 deficiency. Our study underlies the importance of the differential diagnosis between STAT3 and DOCK8 deficiencies in order to improve diagnostic criteria and to propose appropriate therapeutic approaches. In addition, our findings emphasize the role of NGS in detecting mutations that induce overlapping phenotypes.

## 1 Introduction

Hyper-IgE syndromes (HIES) represent a heterogeneous group of inborn errors of immunity (IEI) associated with elevated serum IgE, atopic dermatitis, susceptibility to skin and lung infections along with other non-immunological manifestations in some cases (1). The most common form of HIES is due to loss of function (LOF) mutations in Signal Transducer and Activator of Transcription 3 (*STAT3)* gene (2, 3). Autosomal dominant STAT3 deficiency (AD-STAT3) is a multi-system disorder characterized by severe neonatal eczema, “cold” staphylococcal abscesses, recurrent bacterial respiratory tract infections that may lead to bronchiectasis or pneumatoceles, mucocutaneous candidiasis, eosinophilia, and markedly elevated IgE. Non-immunological features, including a characteristic facial appearance, retention of deciduous teeth, osteopenia, scoliosis, hyper-extensibility, and vascular abnormalities, are key distinguishing features of this disorder (4).

Over the last decade, molecular diagnosis and particularly next‐generation-sequencing (NGS) tremendously contributed to the diagnosis of IEI (5) and consequently helped to unravel the underlying genetic defects of new HIES entities. In 2014, we and others have reported a novel entity due to autosomal recessive (AR) hypomorphic mutations in *Phosphoglucomutase 3 (PGM3)* gene (6, 7). PGM3 deficient patients presented the classical clinical triad of recurrent pneumonia, recurrent skin abscesses, and highly increased serum IgE levels along with connective tissue and bone abnormalities. A distinctive clinical feature in these patients is the severe developmental delay and intellectual disability. These additional features guided us for the genetic study in nine other patients presenting the same clinical phenotype. Indeed, we identified a founder mutation in *PGM3* gene (p.Glu340del) in twelve patients belonging to three apparently unrelated families (8). These findings facilitated the development of preventive approaches through genetic counseling and prenatal diagnosis in consanguineous affected families. In addition, we demonstrated in 2019 that defective glycosylation in PGM3 deficient patients led to reduced gp130 expression and consequently, impaired STAT3 phosphorylation. This may account for the overlapping clinical features shared by PGM3 deficiency and STAT3 deficiency (9).

According to the latest International Union of Immunological Societies Expert Committee (IUIS) classification, eleven defects are recognized as the genetic causes of HIES caused by mutations in ten genes. In addition to AD-LOF STAT3 deficiency, AR PGM3 deficiency, AR LOF IL6ST partial deficiency, AD IL6ST partial deficiency, AR IL6ST complete deficiency, AR IL6R deficiency, AR ZNF341 deficiency, AD ERBIN deficiency, AD TGBFR deficiency (TGFBR1 and TGFBR2) deficiency, AR SPINK5 deficiency and AD CARD11 deficiency are under the umbrella of HIES (10). A Phenotypic classification in patients presenting clinical symptoms identical to the original STAT3-HIES syndrome could help distinguishing the different entities. Indeed, patients with IL-6 signaling defects, including IL6R, IL6ST, ZNF341, and PGM3 deficiencies are phenocopies of STAT3 deficiency. In addition, patients with deficiencies involving IL6ST protein, the co-receptor of IL-6 family cytokines, including AR IL6ST and AR PGM3 deficiency are characterized by psychomotor retardation (11). Although dedicator of cytokinesis 8 (DOCK8) deficiency is no longer classified as a HIES but as a combined immunodeficiency, it still should be considered in patients with a HIES clinical phenotype. This defect due to bi-allelic mutations in *DOCK8* gene is characterized by distinctive clinical features including severe viral infections, neurological complications, increased risk of malignancies, autoimmunity in addition to the clinical triad observed in STAT3 deficiency (12, 13).

High total IgE levels, eosinophilia and atopic symptoms may also be associated with other IEI, including Omenn Syndrome, Wiskott-Aldrich syndrome, WIP deficiency, ARPC1B deficiency, IPEX syndrome, STAT5b deficiency, STK4 deficiency, MALT1 deficiency, IL21 deficiency, and Di-George syndrome making the diagnosis of HIES more difficult (14).

Herein, we report a study conducted in 11 patients presenting with a HIES clinical phenotype. Candidate gene or WES allowed us to identify two mutations in *STAT3* and two deletions in *DOCK8*. The clinical and molecular differential diagnosis between STAT3 and DOCK8 deficiencies is crucial to establish accurate final diagnosis and to develop appropriate therapeutic approaches, especially when patients do not present the typical features of the disease.

## 2 Material and methods

### 2.1 Patients

Nine Tunisian and two Libyan patients with clinical suspicion of Hyper IgE syndrome were investigated at Pasteur Institute of Tunis. Blood samples were collected for immunological and genetic investigations. This study was approved by the local Ethics Committee, and informed consents were obtained at enrollment.

### 2.2 Immunologic investigations

Standard flow cytometric methods were used for staining of cell surface markers. Heparinized venous blood was collected and peripheral blood mononuclear cells (PBMCs) were obtained by Ficoll-Hypaque density gradient centrifugation. PBMCs were incubated with labeled monoclonal antibodies (CD3, CD4, CD8, CD19, CD56, CD16) (BD Biosciences, San Diego) for 30 min at 4°C. After staining, the cells were washed, fixed with 300 μl of phosphate-buffered saline (PBS) containing 1% paraformaldehyde, and analyzed by flow cytometry. As previously described (8), Th17 cells (CD4^+^IL17^+^INFγ^-^) were identified by means of intracellular staining of IL-17. Briefly, adherent monocytes were removed from the PBMC preparation by incubation for 3 h at 37°C and under an atmosphere containing 5% CO2. 2.5 × 10^6^ non-adherent cells were stimulated overnight with 40 ng/ml phorbol 12-myristate 13-acetate (Sigma-Aldrich) and 1 µM ionomycin (Sigma-Aldrich) in the presence of a secretion inhibitor (1 μl/ml GolgiPlug BD Biosciences). CD4^+^ surface-stained cells were fixed, permeabilized (Cytofix/Cytoperm;BD Biosciences), and stained with anti-IL-17A and anti IFN-γ antibodies (BD Biosciences). Stained cells were analyzed on a BD FACS Canto II Flow Cytometer and results were analyzed using Cell Quest Pro software (BD Biosciences). Data were analyzed using FlowJo software.

### 2.3 STAT3 phosphorylation

The analysis of STAT3 phosphorylation was performed as described previously (8). Cells were stimulated with IL-6 (20 ng/mL, R&D Systems, Inc., Minneapolis, MN) for 20 min at 37°C and then fixed at 37°C using Cytofix buffer for 10 min, permeabilized on ice with Perm Buffer III for 30 min (BD Biosciences), stained with phospho-STAT3 (pY705) antibody (BD Biosciences) for 1 h at room temperature, washed again, and re-suspended in FACS buffer for flow cytometry (BD FACScanto II). Data were analyzed using FlowJo software.

### 2.4 Molecular analysis

As previously described, genomic DNA was extracted from peripheral blood mononuclear cells (PBMCs) using the standard phenol-chloroform procedure (8). All *STAT3* (NM_139276.3) gene exons and exon/intron boundaries were amplified by polymerase chain reaction (PCR) with specific primers. PCR amplification of *DOCK8* (NM_203447.4) cDNA and genomic exons (2, 11, 21, 31, 41, 44, 45, 46, 47, and 48) was performed with specific primers. PCR products were purified using the EXO-SAP (Thermo Scientific) cleanup procedure and sequenced with the BigDye Terminator kit V3.1 (Applied Biosystems). Sequencing was performed on an automated sequencer from Applied Biosystems (Applied Biosystems, Foster City, CA).

### 2.5 Western blotting

As previously described (8), total proteins were extracted from Epstein–Barr virus (EBV)-transformed B cell lines. 2 x 10^6^ cells were harvested, washed briefly with ice-cold PBS and lysed in 200µl 1X Laemmli buffer (12,5 mM TrisHCL, 4% glycerol, 0,4% SDS). Cell lysates were heated at 100°C for 5 min, centrifuged at 12,000 rpm for 20 min and then supernatant was used for immunoblotting. Bicinchoninic Acid solution BCA protein quantitation solution (Sigma) was used for protein quantification. 25 µg of total protein per lane were separated on a 10% SDS–PAGE and electroblotted onto polyvinylidene difluoride membrane (PVDF) (GE Healthcare Biosciences). The PVDF membrane was incubated in blocking buffer (TBS 1X, 0.4%Tween-20, 5% non-fat dry milk) for 1 hour at room temperature, probed overnight at 4°C with monoclonal rabbit anti-DOCK8 antibody (1:500, HPA00321, Sigma Aldrich), washed three time with PBS (PBS 1X, 0.4% Tween-20), and incubated with the secondary HRP-conjugated antibody (goat anti-rabbit IgG, 1:2000, Cell Signaling Technology). Rabbit anti-β-actin antibody (1:100, A2103, Sigma Aldrich) was used as an internal control probe. Protein detection was performed by Pierce™ ECL Western Blotting Substrate (Thermo Scientific).

## 3 Results

### 3.1 Clinical and laboratory findings

**Figure 1:**
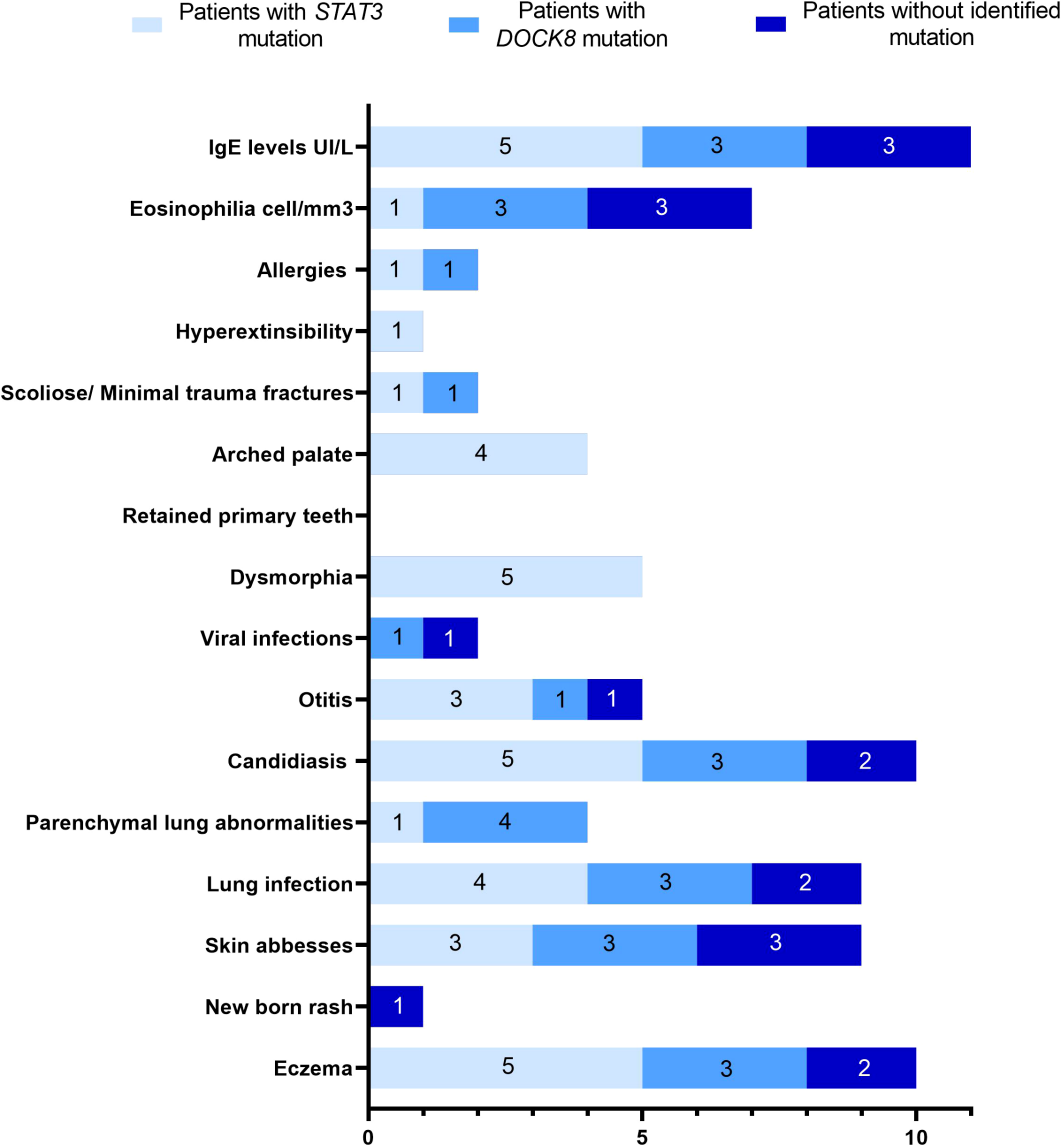
Spectrum of clinical manifestations in eleven HIES suspected patients.

Eleven patients belonging to eight families (including three consanguineous), and presenting with a clinical suspicion of HIES, were investigated in the current study. The detailed clinical phenotype is summarized in **Table 1**. All but one patient presenting with a newborn rash had eczema. The latter was severe in 8/11 patients with papulopustular skin lesions in P5 and lichenified skin lesions in P9. Recurrent lung infections were diagnosed in 10/11 patients leading to parenchymal lung diseases including pneumatocele in P1 and bronchiectasis in three patients (P6, P7, and P8) which contributed significantly to reduced quality of life. Three patients (P1, P2, and P7) had severe manifestations leading to death in one patient (P7) from respiratory failure due to severe bronchiectasis. Ten among 11 patients had a history of chronic mucocutaneous candidiasis (CMC). Connective tissue and bone abnormalities including facial dysmorphism, scoliosis, fracture following minor trauma, retention of deciduous teeth, and hyperextensibility were observed in 5/11 patients. Food allergy were identified in two cases including allergy to chocolate and fried food in P1 and allergy to milk protein in P6 (**Figure 1**).

All patients had markedly increased serum IgE levels (ranged from 850-130000 IU/mL), eosinophilia was present in 7/11 patients and was higher than >1500 cell/mm^3^ in 4 patients (P6, P7, P8, and P11). Absolute lymphocyte counts were performed in 8 patients for whom peripheral blood samples were available (**Supplementary Table1**). P9 had an inverted CD4/CD8 ratio. Memory B cells (CD19^+^CD27^+^) were investigated in 5 patients and were low in all of them. Th17 cell’s percentages (CD4^+^IL17^+^IFNγ^-^) were evaluated in 5 patients and were low in two among them (<0.5%). STAT3 phosphorylation was performed in 10 patients and was impaired in 4 patients (P4, P5, P7, and P8) upon IL-6 stimulation.

### 3.2 Genetic findings in patients with suspected HIES clinical phenotype

The suspicion of STAT3-HIES was based on a modified NIH-HIES scoring system (15). STAT3-HIES was considered highly likely in patients with serum IgE > 1000 IU/ml, and NIH-HIES clinical score> 30. Reduced Th17 cells (< 0.5%) was considered as an additional criterion when available. DOCK8 deficiency was suspected in patients with HIES clinical phenotype and severe viral infection, food allergies, and/or low IgM levels. Patients, for whom Sanger sequencing of *STAT3* or *DOCK8* had not led to a molecular diagnosis, were investigated by NGS (gene panel and/or exome).

#### 3.2.1 STAT3 deficiency

Sequence analysis of *STAT3* gene was performed in 9 patients belonging to 6 kindreds, and two different mutations were identified (**Table 1**).

**Table 1:** Clinical and genetic findings in patients with Hyper IgE Syndromes.

Patient	P1	P2	P3	P4	P5	P6	P7	P8	P9	P10	P11
**kindred**	K1	K1	K1	K1	K2	K3	K4	K5	K6	K7	K8
**Consanguinity**	–	–	–	–	–	+	+	+	–	–	–
**Gender**	M	F	F	M	F	F	M	F	M	F	F
**Country of origin**	Tunisia	Tunisia	Tunisia	Tunisia	Libya	Tunisia	Libya	Tunisia	Tunisia	Tunisia	Tunisia
**Status**	Alive	Alive	Alive	Alive	Alive	Alive	Dead	Alive	Alive	Alive	Alive
**Mutated gene**	*STAT3*	*STAT3*	*STAT3*	*STAT3*	*STAT3*	*DOCK8*	*DOCK8*	*DOCK8*	ND	ND	ND
**Mutation**	c.985A>G	c.985A>G	c.985A>G	c.985A>G	c.1858A>G	Ex1-Ex44 Del	Ex46 Del	Ex1-Ex44 Del	ND	ND	ND
**Amino acid exchange**	p.M329V	p.M329V	p.M329V	p.M329V	p.T620A	ND	G1988fsX1990	ND	ND	ND	ND
**Type of mutation**	Substitution	Substitution	Substitution	Substitution	Substitution	Deletion	Deletion	Deletion	ND	ND	ND
**Zygosity**	Heterozygous	Heterozygous	Heterozygous	Heterozygous	Heterozygous	homozygous	homozygous	homozygous	ND	ND	ND
**Age at genetic diagnosis**	2 years and 4 monthes	2 years and 4 monthes	4 years	40 years	5 years	7 years	2 years after his death	6 years and 6 months	ND	ND	ND
**Eczema**	severe	severe	severe	severe	Severe	severe	moderate	severe	severe	moderate	Absent
**New born rash**	–	–	–	–	–	–	–	–	–	–	+
**Skin abbesses**	+	+	–	–	+	+	+	+	+	+	+
**Lung infection**	+	+	+	+	–	+	+	+	+	+	+
**Parenchymal lung abnormalities**	Pneumatocele	–	–	–	–	Bronchiectasis	Bronchiectasis	Bronchiectasis	–	–	–
**Candidiasis**	+	+	+	+	+	+	+	+	–	+	+
**Otitis**	+	+	–	+	–	–	+	–	–	+	–
**Viral infections**	–	–	–	–	–	Molluscum contagiosum	–	–	–	–	+
**Dysmorphia**	+	+	+	+	Craniosynostosis	–	–	–	–	–	–
Patient	P1	P2	P3	P4	P5	P6	P7	P8	P9	P10	P11
**Retained primary teeth**	–	–	–	–	–	–	–	–	–	–	–
**Arched palate**	+	+	+	+	–	–	–	–	–	+	–
**Scoliosis/Minimal trauma fractures**	–	–	–	+	–	+	–	–	–	–	–
**Hyperextinsibility**	–	–	–	+	–	–	–	–	–	–	–
**Other manifestations**	–	–	–	–	Failure to growth	–	Sclerosing cholangitis	–	Failure to growth, puberty delay, lymphocytosis and lymphoadenopathy	Acute pyelonephritis	–
**Allergies**	–	Food allergy	–	–	–	Food allergy	–	–	–	–	–
**Eosinophilia >700 cell/mm^3^**	–	–	–	–	+	+	+	+	+	+	+
**IgE levels UI/L**	2875	2323	850	2947	1000	5490	13600	7120	113000	3362	3970
**NIH score**	51	41	28	38	ND	46	50	46	35	58	36

ND, Not determined.

**Figure 2:**
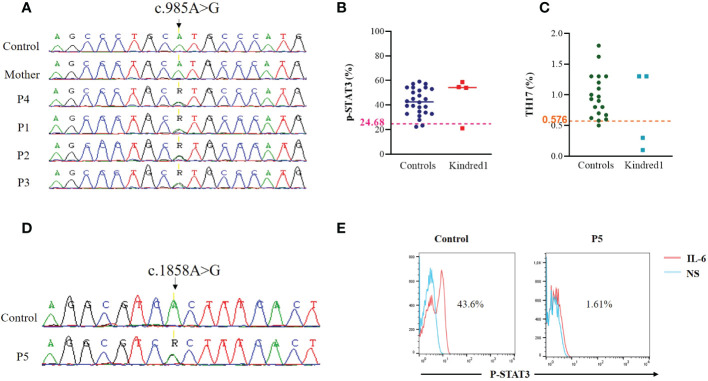
Genetic and functional assessment in STAT3 deficient patients **(A)** Genomic DNA sequence analysis in family K1 showing *STAT3* heterozygous variant (c.985A>G) in patients compared to WT/WT healthy control **(B)** Flow cytometric analyses of STAT3 phosphorylation (Tyr705) in PBMCs stimulated with IL-6 (20 ng/mL) for 20 minutes in family K1. Threshold values were determined according to the 5th percentile (95% confidence interval) in control groups. Significant differences between patients and controls were defined as below to the threshold value **(C)** Percentages of Th17 (CD4^+^IL17^+^INFγ^-^) cells in non adherent PBMCs stimulated with PMA ionomycin for 12 hours, as measured by Flow cytometry in patients from family K1 compared to controls. Horizontal bars indicate medians **(D)** Genomic DNA sequence analysis in P5 showing *STAT3* heterozygous variant (c.1858A>G) in P5 compared to WT/WT healthy control **(E)** Flow cytometric analyses of STAT3 phosphorylation (Tyr705) in EBV transformed cell lines stimulated with IL-6 (20 ng/mL) for 20 minutes in P5.

The first mutation located in the DNA binding domain of *STAT3* (p.M329V; c.985A>G) (SCV002765910) was identified in one father and his three children. The mother was confirmed negative for this mutation (**Figure 2A**). This variant, absent in 50 Tunisian controls and in OMIM and ClinVar databases, was predicted to be deleterious using PolyPhen2 and MutationTaster. STAT3 phosphorylation was slightly affected in the father P4 (21.1%), and normal in his children (**Figure 2B**). Th17 cells were reduced in two children P1 (0.3%) and P3 (0.1%) (**Figure 2C**). Memory B cells, assessed only in P1 and P2, were low. Only one STAT3-deficient patient (P3) had a low NIH clinical score (16) and was diagnosed when her siblings were investigated.

The second mutation is located in the SH2 domain of *STAT3* (p.T620A; c.1858A>G) and was previously reported by Renner et al. (17) (**Figure 2D**). It was identified in one Libyan patient (P5) presenting with a typical clinical phenotype of AD-STAT3 deficiency. Indeed, this patient had a history of papulopustular skin lesions in the face and the neck, recurrent skin abscesses, oral candidiasis, onychomycosis, craniosynostosis, and growth failure. NIH score was not completed due to loss of follow-up. As expected, STAT3 phosphorylation was impaired in the patient’s EBV-B cell lines in response to IL-6 stimulation (**Figure 2E**).

#### 3.2.2 DOCK8 deficiency

When first investigated, P6 was an 8-year-old girl born to consanguineous Tunisian parents. She had a history of recurrent pneumonia and bronchiectasis since the age of 18 months. She suffered from recurrent cutaneous Molluscum contagiosum viral infection, and oral candidiasis. She had high serum IgE levels (5490 IU) and eosinophilia. She also had a food allergy to cow’s milk protein. The clinical phenotype was compatible with a DOCK8 deficiency.

**Figure 3:**
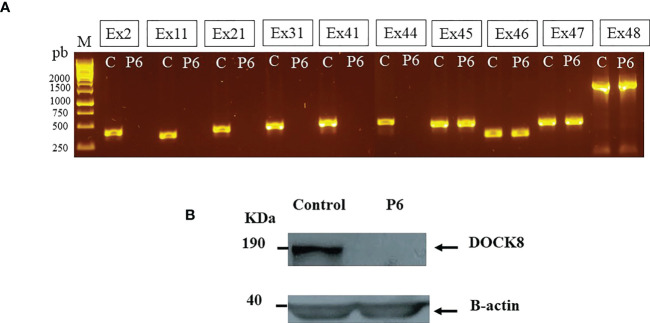
*DOCK8* (Ex1-Ex44) deletion in P6 **(A)** Lack of PCR products from *DOCK8* genomic DNA suggesting exonic deletions up to exon 44 **(B)** DOCK8 protein expression was evaluated in P6 by Western blot in denaturated whole-cell lysates from EBV transformed cell lines, using anti-DOCK8 antibody.

Lymphocyte phenotyping (**Supplementary Table 1**) showed normal percentages of CD3^+^ and CD4^+^ T cells, an increased percentage of CD19^+^CD27^−^ naive B cells as well as decreased percentages of CD19^+^CD27^+^IgD^+^ unswitched memory B cells and CD19^+^CD27^+^IgD^−^ switched memory B cells arguing in favor of DOCK8 deficiency (18). Molecular analysis showed a failure for *DOCK8* cDNA amplification suggesting deletion including part of the *DOCK8* gene (data not shown). The lack of PCR products for exons 2, 11, 21, 31, 41, and 44 is highly suggestive of a homozygous deletion up to exon 44 of *DOCK8* gene (Ex1_Ex44del) (**Figure 3A**). Expression of DOCK8 could not be detected by Western blot in patient’s EBV-B cell lines (**Figure 3B**). At 9 years and 3 months of age, haploidentical related donor HSCT was performed, and a clinical improvement was noted.

**Figure 4:**
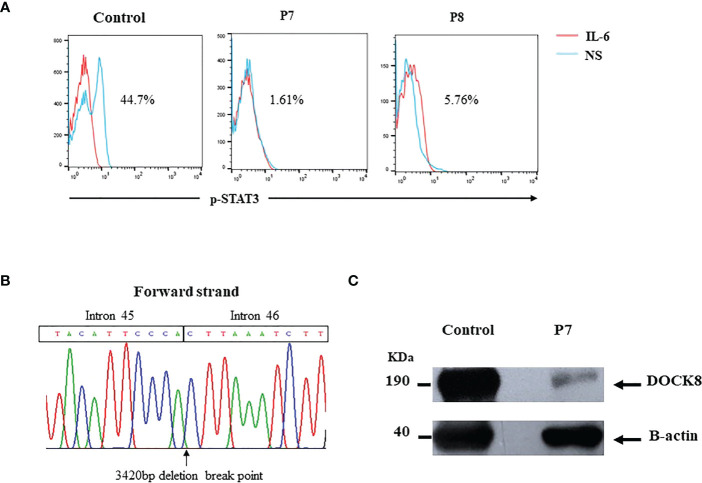
**(A)** Defective STAT3 phosphorylation in DOCK8 deficient patients **(B)** *DOCK8* Ex46 deletion in P7. Chromatogram showing the 3420bp deletion breakpoint in the patient’s genomic DNA **(C)** DOCK8 protein expression in P7 showing residual expression.

P7 was a 12-year-old boy born to consanguineous Libyan parents. He had a history of a sister who died at 6-year-age from severe infections. He was diagnosed with eczema, recurrent infections, bronchiectasis, staphylococcal skin abscesses, otitis, and oral candidiasis. Sclerosing cholangitis (SC) was reported later. Laboratory investigations showed elevated IgE, eosinophilia, low percentages of CD4^+^ T cells, and low IgM levels. STAT3 phosphorylation was highly affected in the patient (1.6%) (**Figure 4A**). Unfortunately, the patient died at 14 years old from severe respiratory infections before definitive diagnosis. Whole exome sequencing (WES) revealed a homozygous 3420pb *DOCK8* genomic deletion (IVS45+792_IVS46+2020del) (SCV002765912), including exon 46 (EX46del) (**Figure 4B**). At the protein level, this deletion has created a stop codon at position 1990 (G1988fsX1990). Western blot analysis revealed residual levels of full-length DOCK8 protein in the patient’s lymphocytes (**Figure 4C**).

P8 was a 4-year-old girl born to consanguineous Tunisian parents. She presented with severe eczema, recurrent skin abscesses, recurrent pneumonia, bronchiectasis, and oral candidiasis. Laboratory investigations showed elevated IgE, eosinophilia, and low IgM levels. Lymphocyte phenotyping showed normal percentages of CD3^+^ and CD4^+^ T cells, and decreased memory B cells. Initially, STAT3-HIES was suspected in this patient due to HIES clinical phenotype (NIH score 46) in the absence of severe viral infection and food allergy. STAT3 phosphorylation was reduced in this patient (5.18%) (**Figure 4A**), and Sanger sequencing of *STAT3* gene did not reveal any mutation. WES revealed the presence of the same large deletion in *DOCK8* gene (Ex1_Ex44del), identified in P6. This patient will shortly benefit from HSCT.

#### 3.2.3 HIES suspected patient without genetic diagnosis

As a first-line genetic screening strategy, *STAT3* candidate gene was sequenced in the remaining patients clinically diagnosed with HIES (P9, P10, and P11), and no mutation was identified. STAT3 phosphorylation was assessed as well, and was normal in these patients (**Supplementary Figure 1**). P9 who had IgE levels of >100.000 IU/l and marked eosinophilia did benefit from a targeted gene sequencing, using “Invitae Primary Immunodeficiency Panel” including 407 genes, and no pathogenic variant was identified. The same strategy was applied in P10 who had the highest NIH clinical score of 58 points, and panel gene sequencing was performed but was not successful. Subsequently, WES has been applied for patients P9, P10, and P11. A duo analysis was performed for P9, and a trio analysis for P10 and P11 but no pathogenic variant could be identified.

## 4 Discussion

The evolving definition of HIES in recent years since the discovery of the prototype of these disorders more than 50 years ago (19) made the diagnosis of HIES more challenging. AD-STAT3 deficiency due to LOF dominant negative mutations in *STAT3*, identified in 2007, is considered as the major molecular etiology of HIES worldwide (2, 3). Ten other molecular etiologies have been identified owing to the progress made in genetic testing. According to the latest updated IUIS classification of Human Inborn Errors of Immunity (IEI), two novel entities were recognized as genetic causes of HIES, ie AD IL6ST partial deficiency and AR IL6ST complete deficiency (10). DOCK8 deficiency initially described as the AR form of hyper-IgE syndrome (20) is now classified as a combined immunodeficiency, generally less profound than SCID, but still shares some clinical features with STAT3 deficiency adding to the complexity of the differential diagnosis of HIES with other IEI (21, 22).

In the present study, eleven patients with suspected HIES clinical phenotype were investigated. Except P5, completed NIH-HIES scoring system was available for all the patients. Candidate gene approach was applied as a first line screening strategy. Indeed, *STAT3* gene was sequenced when STAT3-HIES was considered highly likely according to a modified NIH-HIES scoring system (15). This strategy allowed us to identify two *STAT3* mutations in two families.

The first mutation (p.M329V) located in the DNA binding domain of *STAT3* did not alter STAT3 phosphorylation which is consistent with previous studies (4). This novel variant was predicted to be deleterious using PolyPhen2 and MutationTaster, and was first identified in three children and later in their father. The suspicion of HIES made by the referring pediatrician did not concern the father who remained undiagnosed until adulthood probably due to the lack of awareness preventing PIDs diagnosis30years ago (23). Physical examination, at the time of referral, revealed a characteristic face with a broad nasal bridge and a prominent nose, and joint hyperextensibility. During his childhood, the father suffered from eczema, lung infections, candidiasis, minor trauma fractures, and scoliosis. A major finding in this family is the variability of the clinical and immunological phenotype despite the presence of the same mutation. Indeed, the twins (P1 and P2) had the highest NIH-HIES score due to their severe clinical phenotype, and were therefore referred by their pediatrician for HIES suspicion. Percentages of Th17 cells varied as well in this family. P2 had normal percentages of Th17 cells despite having onychomycosis and oral candidiasis suggesting that other factors could be responsible for susceptibility to fungal infections. These findings are in accordance with previous studies related to variable clinical and immunological manifestations of *STAT3* mutations in patients among the same family (2, 24). Epigenetic mechanisms, environmental factors or genetic mosaicism could account for such variability (24, 25). The second mutation located in the SH2 domain of *STAT3* was previously reported by Renner et al. (17). As expected, STAT3 phosphorylation was impaired in response to IL-6 stimulation. While NIH score was not completed for this patient, her clinical phenotype was highly suggestive of STAT3 deficiency, and candidate gene strategy was successful.

Candidate gene strategy was also applied in P6 for whom DOCK8 deficiency was suspected according to already established criteria (26). This patient presented with a HIES clinical phenotype in addition to a severe viral infection, food allergy, in the absence of parenchymal lung abnormalities, minimal trauma fracture, and retained primary teeth. In addition, laboratory findings showed increased IgE serum levels, particularly high eosinophilia, and low switched and unswitched memory B cells. Such clinical and biological findings have been reported to be highly suggestive of DOCK8 deficiency and could guide molecular testing (26, 27). Indeed, since *DOCK8* is a large gene containing 48 exons that span over 200 kilobases (28), such an approach is important to reduce molecular diagnosis costs in resource-limited-settings. Accordingly, we have been able to identify a large deletion up to exon 44 of *DOCK8* gene (Ex1_Ex44del) by PCR targeting gene exons. Western blot analysis demonstrated the absence of DOCK8 protein. The patient had undergone successful HSCT after molecular confirmation of diagnosis. Eczema, and Molluscum contagiosum lesions did resolve rapidly after transplantation. This was already reported in a large international cohort of DOCK8-deficient patients published in 2019 by Aydin SE et al. (16), confirming that HSCT represents the only curative treatment for DOCK8 deficiency. The same mutation was identified by WES in P8, from an unrelated family, initially suspected to have STAT3 deficiency. Indeed, P8 had elevated NIH-HIES clinical score of 46 points, and had no severe viral infections nor allergies. STAT3 phosphorylation was shown to be reduced in this patient, but Sanger sequencing of *STAT3* gene did not reveal any mutation. Thus, WES was applied and revealed the same deletion as in P6. This deletion has been published earlier by Engelhardt et al. (26). Interestingly, these two unrelated families are originating from the same region of Tunisia suggesting a potential founder effect. Indeed, particular mutant alleles are enriched in certain geographical areas (13). Homozygosity mapping to search for homozygous haplotypes, using high-density oligonucleotide arrays, could confirm this hypothesis. One clinical implication of the existence of a founder effect is the implementation of preventive approach through genetic counseling in areas with high incidence of consanguinity. Another interesting observation with regard to the same identified *DOCK8* deletion is the variability of the clinical phenotype. P6 presented with typical features of severe cutaneous viral infections, atopic dermatitis, recurrent respiratory infections, and food allergy. However, P8 presented with HIES clinical phenotype and absence of severe viral infections and allergies. With regard to P7 who is a Libyan patient, initially presenting with eczema, recurrent infections, bronchiectasis, staphylococcal skin abscesses, otitis, and oral candidiasis. Laboratory findings revealed increased IgE levels, and eosinophilia. He had a NIH-HIES score of 46 points. STAT3 deficiency was suspected at that time. STAT3 phosphorylation was impaired as assessed by flow cytometry. Sclerosing cholangitis was reported later and further immunological investigation revealed low percentages of CD4^+^ T cells and low serum IgM levels. WES was performed and revealed the presence of a homozygous deletion in *DOCK8* gene including exon 46 (IVS45+792_IVS46+2020del). This mutation was previously reported by Engelhardt et al. (13). Since exon 46 was absent in the patient’s cDNA, the authors concluded that the patient harbored a homozygous genomic deletion of exon 46. We showed herein that exon 46 deletion is due to a homozygous 3420pb *DOCK8* genomic deletion. This frameshift mutation led to a premature stop codon at position 1990. Residual level of full-length DOCK8 protein was detected in the patient’s lymphocytes probably due to nonsense-mediated mRNA decay mechanism in the case of premature stop codons (29). Unfortunately, the patient died from severe respiratory infections before HSCT. His clinical phenotype had some overlap with STAT3 deficiency, and did not present with hallmarks of DOCK8 deficiency including allergic diseases and cutaneous viral infections. This may have contributed to the diagnostic delay. Sclerosing cholangitis, a rare symptom, reported in only 5% of a large series including 136 DOCK8 deficient patients was observed in this patient (30).

The remaining patients, for whom Sanger sequencing of candidate genes were unsuccessful, did undergo IEI genes panel and/or WES performed with the support of “Jeffrey Modell Foundation” and “The Asian Primary Immunodeficiency (APID) Network” respectively. Unfortunately, no rare variant could be identified in P9, P10, and P11. Negative findings in these patients could be attributed to incomplete exome capture or low coverage of intron-exon boundaries leading to false-negative results (5).The diagnostic yield of undiagnosed PID patients using NGS, varied considerably from 15 to 79% depending on patient recruitment, parental consanguinity rates, and PID phenotype subgroups (31). Two among five patients reached definitive genetic diagnosis using WES leading to a diagnostic yield of 40%. Remaining patients presenting with symptoms suggestive of PIDs will benefit from a whole genome sequencing (WGS). Indeed, P9 had recalcitrant eczema, recurrent skin abscesses, failure to growth, puberty delay, lymphocytosis, and lymphadenopathy. He also had elevated serum IgE (>100.000 IU/l) and marked eosinophilia (2750/mm^3^). P10, who had the highest NIH clinical score, presented with eczema, recurrent skin abscesses, recurrent pneumonia, oral candidiasis, recurrent otitis, severe pyelonephritis, and high arched palate. She also had elevated serum IgE (3362 IU/l) and marked eosinophilia (4000/mm^3^). P11 had newborn rash, recurrent skin abscesses, cutaneous viral infection, pneumonia, and oral candidiasis.

The updated classification of inborn errors of immunity published in 2022 reported 485 genetic defects including 10 HIES-causing candidate genes (10). Although not classified among the latters, DOCK8 deficiency shares several clinical features with STAT3 deficiency and other HIES. While candidate gene sequencing still one of the key diagnostic tools in our settings for IEI patients with typical clinical and immunological phenotype, it is time- and cost-consuming especially when multiple genes are involved. Our approach in the present study was based on clinical features and suggestive laboratory findings, which guided us to candidate gene sequencing as a first line screening strategy. This approach was successful in 3 families with a typical clinical phenotype, in which we achieved a definitive diagnosis. Nevertheless, in two DOCK8 deficient patients, STAT3-HIES was much more suspected than DOCK8 deficiency, initially. The latter patients did not present the typical clinical phenotype of DOCK8 deficiency and STAT3 phosphorylation was impaired. WES allowed us to correct the diagnosis in these two patients and one will shortly benefit from HSCT. Although STAT3 phosphorylation assay could be an important functional assay in HIES STAT3-dependent pathway, but could be normal in a significant number of patients (32). In addition, it could also be impaired in DOCK8 deficient patients (33). Keles and collaborators showed that STAT3 phosphorylation is decreased in DOCK8 deficient patients. This decrease is not dependent on the IL-6 receptor complex, but is in a Guanine exchange factor (GEF) dependent manner. Indeed, DOCK8, GEF involved in actin cytoskeleton regulation, activates Rho GTPase proteins including Rac1, Cdc42, and RhoA. Cdc42 activates downstream kinases involved in STAT3 activation (34). This DOCK8-dependent upregulation of STAT3 might explain the overlap between DOCK8 deficiency and STAT3 deficiency (33).

In summary, this study highlights the challenge of the differential diagnosis between STAT3 and DOCK8 deficiencies in patients with atypical clinical phenotype. As previously mentioned, some clinical features observed in DOCK8 deficiency may overlap with those of STAT3 deficiency. In addition, clinical phenotype of DOCK8 deficiency may be significantly variable including for the same mutation with features, which may not include the typical viral infections and the food allergy. This differential diagnosis challenge could be overcome through an appropriate and combined strategy including clinical, immunological and molecular investigations. As the majority of DOCK8 deficient patients lack DOCK8 protein expression, the investigation of DOCK8 expression by flow cytometry will help distinguishing between DOCK8 deficiency and STAT3 deficiency (35). Engelhardt et al, proposed a modified HIES score named “DOCK8 score” that could assist physicians to distinguish a patient with a DOCK8 deficiency from one with a STAT3 deficiency. The authors used a linear classifier based on five DOCK8-relevant features, including parenchymal lung abnormalities, eosinophilia, sinusitis/otitis, retained primary teeth, and fracture with minor trauma (26).

In conclusion, we present herein 11 patients with HIES clinical phenotype including 6 typical cases for whom candidate gene strategy allowed us to reach a definitive diagnosis in 5 patients with *STAT3* mutations and one patient with a large *DOCK8* deletion. NGS strategy in remaining patients allowed to unmask atypical presentations of DOCK8 deficiency in two patients. Since ten genes are now recognized to be responsible for HIES (10), a customized gene panel could help establish a HIES diagnosis and determine the specific etiology. This panel should include the ten HIES genes, *DOCK8* gene, *TYK2* gene, genes that could be responsible for atypical clinical presentations (36), and genes associated with moderate to severe refractory eczema and elevated Immunoglobulin E (14, 37).

## Data availability statement

The original contributions presented in the study are publicly available. This sequencing data can be found here: Clinvar database, accession numbers (SCV002765910) STAT3 (SCV002765912) DOCK8.

## Ethics statement

The studies involving human participants were reviewed and approved by Biomedical Ethics Committee, Pasteur Institute of Tunis. Written informed consent to participate in this study was provided by the participants’ legal guardian/next of kin.

## Author contributions

RY, NM and MB-A collected the patients’ samples, performed experiments, analyzed the data, wrote and read the manuscript. NM, IB-M, M-RB and MB-A contributed to the conception and design of the study, performed interpretation of results, wrote and read the manuscript. LB-K contributed to the immunological and molecular investigations of the patients. AB, IBF, JA, AH, HT, LB, MD, SH, and MO provided clinical information/were the clinicians in charge of patient care and management. KC performed the genetic analysis. YL provided critical discussion. All authors contributed to the article and approved the submitted version.

## Supplementary material

Supplementary Figure 1 STAT3 phosphorylation in HIES suspected patients (P9, P10, and P11). Flow cytometric analyses of STAT3 phosphorylation (Tyr705) in PBMCs stimulated with IL-6 (20 ng/mL) for 20 minutes in P9, P10, and P11. Threshold values were determined according to the 5th percentile (95% confidence interval) in control groups.

Supplementary Table 1 Lymphocytes subsets and immunoglobulin levels in HIES patients.
