# Supplementary figures and images for: Diagnostic challenge in a series of eleven patients with hyper IgE syndromes
Source: Front Immunol. 2023 Jan 10;13:1057679. doi: 10.3389/fimmu.2022.1057679 (PMC9871884; doi:10.3389/fimmu.2022.1057679)

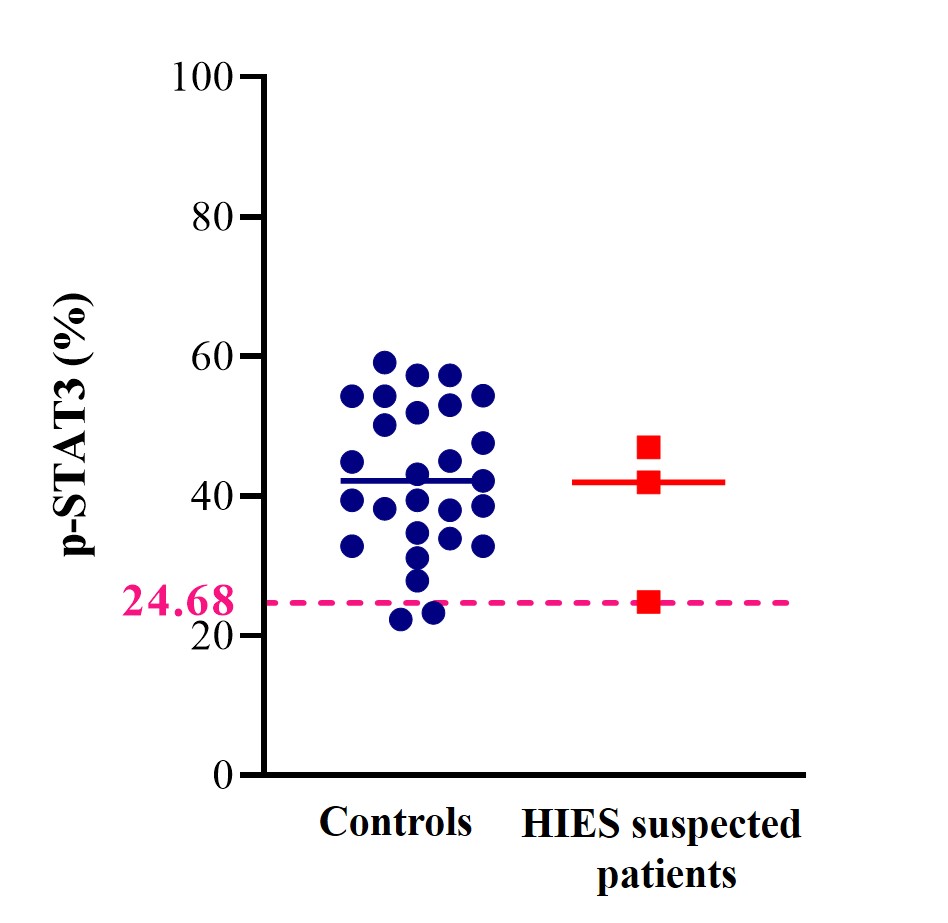

Supplement: Supplementary Figure 1 — STAT3 phosphorylation in HIES suspected patients (P9, P10, and P11). Flow cytometric analyses of STAT3 phosphorylation (Tyr705) in PBMCs stimulated with IL-6 (20 ng/mL) for 20 minutes in P9, P10, and P11. Threshold values were determined according to the 5th percentile (95% confidence interval) in control groups. [file Image_1.jpeg]
