# Supplementary material for: Diagnostic challenge in a series of eleven patients with hyper IgE syndromes
Source: Front Immunol. 2023 Jan 10;13:1057679. doi: 10.3389/fimmu.2022.1057679 (PMC9871884; doi:10.3389/fimmu.2022.1057679)
Supplement: Supplementary Table 1 — Lymphocytes subsets and immunoglobulin levels in HIES patients. [file DataSheet_1.pdf]

Supplementary Table 1:

	P1	P2	P5	P6	P8	P9	P10	P11
**Total lymphocytes (cells/µL)**	4540 (1400-5500)	3800 (1400-5500)	11910 (1400-5500)	4300 (1200-4700)	3310 (1400-5500)	4610 (1400-4200)	3900 (1400-5500)	4210 (1400-5500)
**$CD3^+$ T cells**	59.4 (56-75)	77.5 (56-75)	75.2 (56-75)	49 (60-76)	58.5 (56-75)	71 (56-84)	77.5 (56-75)	42.5 (53-75)
**$CD4^+$ T cells**	36.9 (28-47)	47.5 (28-47)	46 (28-47)	34 (31-47)	34 (28-47)	30.5 (32-51)	48 (28-47)	25.5 (32-51)
**$CD8^+$ T cells**	19.2 (16-30)	26.5 (16-30)	23.5 (16-30)	17.5 (18-35)	26 (16-30)	32 (14-30)	24 (16-30)	17 (14-30)
**$CD19^+$ B cells**	20 (14-33)	2 (14-33)	17 (14-33)	41 (13-27)	17 (14-33)	6.5 (06-23)	17.5 (14-33)	28 (16-35)
**Naïve B cells ($CD27^-$ IgD^+)**	-	-	-	92.5 (51-84)	88.5 (63-86)	-	-	-
**Memory B cells ($CD27^+$ $CD19^+$)**	1.5 (7-24.3)	2.8 (7-24.3)	-	1 (8.1-33.3)	0.7 (7-24.3)	-	4.5 (7-24.3)	-
**Non-switched memory B-cells ($CD27^+$ IgD^+)**	-	-	-	1.1 (5-14)	1.4 (4–16)	-	-	-
**Switched memory B-cells ($CD27^+$ IgD^-)**				1.6 (5-16)	2.7 (4-16)			
**CD4/CD8 ratio**	1.92	1.79	1.95	1.94	1.3	0.95	2	1.5
**NK cells %**	19 (04-17)	18.2 (04-17)	7.3 (04-17)	7 (04-17)	18.7 (04-17)	15.9 (03-22)	3.8 (04-17)	20.7 (03-15)
**Immunoglobulins g/L**								
**IgG**	10.31 (4.82-12.00)	10.35 (4.82-12.00)	13.13 (4.82-12.00)	18.77 (6.46-14.51)	13.59 (5.53-13.07)	13.78 (5.18-14.47)	10.99 (5.53-13.07)	13.82 (4.82-12.00)
**IgA**	1.17 (0.22-1.18)	0.76 (0.22-1.18)	0.602 (0.22-1.18)	2.61 (0.57-2.04)	1.13 (0.33-1.80)	0.95 (0.23-1.37)	1.27 (0.33-1.8)	1.01 (0.22-1.18)
**IgM**	0.84 (0.542.09)	1.8 (0.542.09)	2.002 (0.542.09)	0.87 (0.44-2.42)	0.39 (0.56-2.18)	0.69 (0.42-2.12)	1.01 (0.56-2.18)	1.13 (0.54-2.09)
